# Persistence of self-recruitment and patterns of larval connectivity in a marine protected area network

**DOI:** 10.1002/ece3.208

**Published:** 2012-02

**Authors:** Michael L Berumen, Glenn R Almany, Serge Planes, Geoffrey P Jones, Pablo Saenz-Agudelo, Simon R Thorrold

**Affiliations:** 1 Red Sea Research Center, King Abdullah University of Science and TechnologyThuwal, 23955, Kingdom of Saudi Arabia; 2 Biology Department Woods Hole Oceanographic InstitutionWoods Hole, Massachusetts 02540; 3 ARC Centre of Excellence for Coral Reef Studies, and School of Marine and Tropical Biology, James Cook University TownsvilleQueensland, 4811, Australia; 4 USR 3278 CNRS EPHE Center de Recherches Insulaires et Observatoire de l'Environnement (CRIOBE) BP 1013 Papetoai98729 Moorea, French Polynesia; 5 Laboratoire d'excellence “CORAIL”BP 1013 Papetoai, 98729 Moorea, French Polynesia

**Keywords:** *Amphiprion percula*, *Chaetodon vagabundus*, connectivity, larval dispersal, marine protected areas, microsatellite parentage analysis, self-recruitment

## Abstract

The use of marine protected area (MPA) networks to sustain fisheries and conserve biodiversity is predicated on two critical yet rarely tested assumptions. Individual MPAs must produce sufficient larvae that settle within that reserve's boundaries to maintain local populations while simultaneously supplying larvae to other MPA nodes in the network that might otherwise suffer local extinction. Here, we use genetic parentage analysis to demonstrate that patterns of self-recruitment of two reef fishes (*Amphiprion percula* and *Chaetodon vagabundus*) in an MPA in Kimbe Bay, Papua New Guinea, were remarkably consistent over several years. However, dispersal from this reserve to two other nodes in an MPA network varied between species and through time. The stability of our estimates of self-recruitment suggests that even small MPAs may be self-sustaining. However, our results caution against applying optimization strategies to MPA network design without accounting for variable connectivity among species and over time.

## Introduction

The challenges associated with measuring larval dispersal in marine organisms with a pelagic larval phase have limited our ability to effectively manage fisheries (Cowen et al. 2006; Botsford et al. 2009) and conserve biodiversity (Jones et al. 2007; Almany et al. 2009a). Marine protected areas (MPAs) have been widely advocated and implemented as a tool that can simultaneously achieve both fisheries management and biodiversity conservation objectives (Roberts et al. 2001; Russ 2002; McCook et al. 2009, 2010; Gaines et al. 2010). However, achieving both of these goals is contingent upon two key, and largely untested, assumptions: that larvae produced within an MPA contribute to populations both within and outside that MPA (Russ 2002).

Long-term persistence of populations within an MPA is facilitated by some degree of self-replenishment (Hastings and Botsford 2006; Jones et al. 2007; Kaplan et al. 2009). As in our previous work, we define “ self-recruitment” at a given location as the proportion of the total number of recruits sampled at that location that are identified as the offspring of adults from that same location. Notably, this is distinct from “local retention” at a location, which is defined as the proportion of larvae that return to their natal origin relative to the total number of larvae produced from that same location (Botsford et al. 2009). Significant levels of self-recruitment may be particularly important for single, isolated MPAs for which there are no other reliable sources of larvae. Increasingly, management agencies are moving to implement networks of MPAs. One key feature of a network is that larval dispersal has the potential to connect MPAs, and such connectivity enhances the resilience of the network (Kaplan et al. 2009; Planes et al. 2009). For example, if populations within an MPA are reduced by a disturbance, larval dispersal from other MPAs can facilitate their recovery. Understanding larval connectivity is therefore key to determining the optimal locations of and spacing between MPA nodes to ensure they are connected by larval dispersal. However, the general lack of any empirical data on larval dispersal means that most MPAs and MPA networks are designed using “best guess” estimates concerning MPA size, location, and spacing (McCook et al. 2009).

A number of different approaches have been used to estimate larval dispersal in marine environments (Thorrold et al. 2002). Coupled biophysical models (Cowen et al. 2006; Paris et al. 2007;Cowen and Sponaugle 2009), otolith (ear bone) chemistry (Elsdon et al. 2008), population genetics (Palumbi 2003; Levin 2006), and surveys of juvenile densities in relation to reserve boundaries (Pelc et al. 2010) have all been used to predict natal origins or dispersal pathways of larvae. However, these methods often rely on untested assumptions, or lack the temporal or spatial resolution to be usefully applied to the design of MPA networks. New methods of larval tagging and genetic parentage analysis overcome some of these obstacles and are beginning to provide the first unequivocal empirical measurements of larval dispersal. Significant levels of self-recruitment have been reported in several benthic spawning (*Pomacentrus amboinensis, Amphiprion percula,* and *A. polymnus*) and pelagic spawning (*Chaetodon vagabundus*) reef fishes using larval tagging methods (Jones et al. 1999, 2005; Almany et al. 2007). More recently, larval dispersal distances have been estimated from microsatellite DNA parentage analysis for *A. percula* (Planes et al. 2009) and *A. polymnus* (Saenz–Agudelo et al. 2009), and a single pelagic spawning species, the surgeonfish *Zebrasoma flavescens* (Christie et al. 2010a). Nonetheless, with the exception of Almany et al. (2007), all these studies represent data for a single species at one time. It is, therefore, difficult to draw any conclusions concerning the generality of dispersal patterns.

The primary goal of our study was to compare temporal patterns of self-recruitment within and connectivity among MPAs for two coral reef fishes with contrasting life histories. We used genetic parentage analysis to determine natal origins of larvae of a pelagic spawning butterflyfish with a relatively long planktonic larval duration (PLD) (*C. vagabundus*) and a benthic-spawning anemonefish with a much shorter PLD (*A. percula*) at three MPAs within a larger MPA network. The three study sites are part of a MPA network designed by The Nature Conservancy in conjunction with local communities in Kimbe Bay, Papua New Guinea (Green et al. 2009). These study species provide a contrast of reproductive strategy, and both taxa are highly targeted by collectors for the aquarium fish trade (Chan and Sadovy 2000). Finally, each species has been the subject of previous studies at this site (Almany et al. 2007; Planes et al. 2009), allowing us to assess the temporal consistency of self-recruitment and connectivity patterns. We predicted that the pelagic spawning species with a longer PLD would exhibit greater connectivity than the benthic spawning species with a shorter PLD.

## Materials and Methods

### Study species

*Amphiprion percula* is a benthic spawning anemonefish (Pomacentridae). In Kimbe Bay, it has a pelagic larval duration (PLD) of approximately 10–13 days (Almany et al. 2007; Berumen et al. 2010). In our study area, *A. percula* obligately uses two host anemones, *Stichodachtlya haddoni* and *Heteractis magnifica*. A strong social hierarchy and high fidelity to the host anemone drives a protandrous reproductive system in which the largest fish in an anemone is the reproductive female and the next largest fish is the reproductive male (Fricke 1979); other smaller fish are immature, nonreproductive males. Eggs are laid in a clutch near the host anemone and are guarded by the parents, and larvae are capable of swimming upon hatching (Fisher et al. 2000). New recruits settle directly to anemones.

*Chaetodon vagabundus* is a pelagic spawning butterflyfish (Chaetodontidae) with a PLD of approximately 38 days (range: 29–48) (Almany et al. 2007). Adults typically form monogamous pairs that are stable for at least several years, and spawning is believed to occur in pairs just prior to dusk (Tanaka 1992). New recruits are found in a narrow range of habitats in the intertidal and subtidal zones on islands with fringing reef and then undergo an ontogenetic shift to a broad range of reef habitats (Pratchett et al. 2008).

### Study site

**Figure 1:**
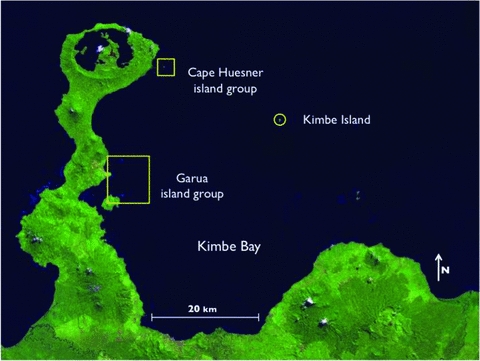
LANDSAT image of study locations in Kimbe Bay, Papua New Guinea.

Our study was conducted in Kimbe Bay, Papua New Guinea. We targeted several sites with substantial habitat on islands with fringing reef within the western half of the bay (Fig. 1). These sites are part of a partially implemented MPA network that spans the length and breadth of Kimbe Bay (Green et al. 2009). Sampling was focused on small islands due to the high abundance of study species and suitable habitat. The discrete nature of these islands allowed for easy definition and measurement of populations.

Sampling locations were within three general areas: (1) Garua, (2) Cape Heusner, and (3) Kimbe Island. The Cape Heusner group consists of two primary islands (Tuare and Kapepa) with significant populations of anemones and *A. percula*. The Garua group consists of four small islands (Restorf, Schumann, Big Malu Malu, and Little Malu Malu). Kimbe Island is a relatively small island (0.19 km^2^) surrounded by several distinct reef habitats, with a total reef area of 0.47 km^2^. The Garua group and Cape Heusner group are separated from Kimbe Island by approximately 33 km and 25 km, respectively. Although there are reefs in other parts of western Kimbe Bay, there are no other sites as suitable for either *A. percula* or *C. vagabundus*.

### Adult collections

Adults of both species were sampled in February 2007 at Kimbe Island. Divers on scuba and snorkel searched all suitable *A. percula* habitat on Kimbe Island and mapped the locations of anemones. Adults were captured using hand nets and clove oil (an anesthetic). Individuals were measured using calipers underwater. Small pieces of fin tissue were taken in situ from the caudal fin and preserved in 85% ethanol in individual 2.0-mL vials. Sampled fish were then returned to their host anemone.

Divers captured adult *C. vagabundus* using barrier nets and hand nets. As with *A. percula*, a small piece of fin tissue was removed from each adult in situ (in this case the posterior soft dorsal) and preserved in 85% ethanol in 2.0-mL vials. Sampled adults were released at the point of capture. The conspicuous fin clip ensured that the same fish were not resampled during the study period, but to aid visual censuses to estimate total population size, each adult was tagged with a 25-mm long external t-bar anchor tag (Floy® FD-94, manufactured by Floy Tag, Seattle, Washington, USA) using a tag applicator (Floy® Mark III). Such tagging has been shown to have no detectable influence on the behavior of this species (Berumen and Almany 2009). To estimate the proportion of the total adult *C. vagabundus* population that was sampled, 108 50 × 10 m visual transects were conducted at the end of the sampling period, with transects distributed among the various habitat types (Table 1). Habitat types at Kimbe Island were ground truthed by snorkelers and subsequently digitized using ArcGIS 9.2 (ESRI) at a scale of 1:4000 using 1-m resolution satellite imagery (IKONOS) to estimate the area of each reef habitat. Stratified transects and area estimates of each habitat allowed us to calculate a total adult population estimate for Kimbe Island following McCormick and Choat (1987).

**Table 1:** Calculations used to obtain population and variance estimates (following McCormick and Choat 1987) for *Chaetodon vagabundus* based on 50 × 10 m visual transects stratified in several habitat types on Kimbe Island. *N_h_* is the number of possible transects that could be fit into each stratum. *W_h_* is the proportion that each strata makes up of the total area. *n_h_* is the number of transects in each stratum. 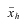 is the mean density of *C. vagabundus* in each stratum. *s_h_*^2^ is the variance around the mean density within each stratum

Strata	Area (m^2^)	Percent area	*N_h_*	*W_h_*	*n_h_*	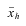	*s_h_*^2^	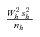	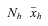	Percent fish
Back lagoon	30,341	6.5	60.7	0.06471	10	0.4000	0.489	2.05 × 10^−4^	24.27	1
Crest	111,744	23.8	223.5	0.23831	54	1.8519	3.600	3.79 × 10^−3^	413.87	25
Flat	235,448	50.2	470.9	0.50212	13	2.0769	4.744	9.20 × 10^−2^	978.01	58
Inshore	11,290	2.4	22.6	0.02408	27	1.4074	1.405	3.02 × 10^−5^	31.78	2
Lagoon slope	80,082	17.1	160.2	0.17079	4	1.5000	3.667	2.67 × 10^−2^	240.25	14
Totals	468,905		937.8	1.00000	108			0.122757	1688	

### Juvenile collections

Juveniles of our two study species were sampled in April 2007 from all islands within our three study areas. Divers on scuba and snorkel searched all known anemones. All *A. percula* recruits and sub-adults were captured using hand nets and a clove oil mixture as above. Fish were measured using calipers. Where possible, small pieces of fin tissue were taken in situ from the caudal fin and preserved in 85% ethanol in individual 2.0-mL vials and fish were retuned to their anemone. For some of the smallest recruits (e.g., < 3 cm), the whole fish was taken. A 3 cm *Amphiprion* recruit is approximately three to four months old (Ochi 1986), and our collection (0.5–3.3cm) likely represents multiple recruitment cohorts (see also Srinivasan and Jones 2006). For the purposes of this study and comparison to previous data, however, we consider the collection as one single cohort for 2007.

Snorkelers searched all suitable habitat for recruits of *C. vagabundus*, which were captured using hand nets and clove oil, measured using calipers, and preserved in 85% ethanol.

### Parentage analysis

DNA from all collected fish was extracted and polymorphic microsatellite loci were screened. Microsatellite screening was performed by Matis– Prokaria (Iceland) using ABI capillary technology for *A. percula*, and at the Université de Perpignan (France) using a Beckman sequencer for *C. vagabundus*. Sixteen microsatellite DNA loci were screened for *A. percula* and 15 microsatellite DNA loci were screened for *C. vagabundus* (Almany et al. 2009b); all loci for both species satisfied Hardy–Weinberg equilibrium assumptions. We used the FAMOZ platform to determine parent–offspring relationships (Gerber et al. 2003; Planes et al. 2009).

## Results

### 2007 Adult collections

We collected a total of 535 adult *A. percula* from 276 anemones (approximately 95% of the population, see Planes et al. (2009)) and 374 adult *C. vagabundus* from Kimbe Island. A total of 175 *C. vagabundus* were recorded in stratified visual survey transects in habitats around Kimbe Island, resulting in a total population size estimate of 1688 (±310 95% CI) adults (see Tables 1 and 2). A sample of 374 adults therefore represented 22.1% of the adult population (95% CI = 18.7–27.1%).

**Table 2:** Summary of population and variance estimates for *Chaetodon vagabundus* on Kimbe Island, calculated following McCormick and Choat (1987). *s*^2^(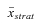) represents the variance of overall stratified mean density. *t_0.05_* is Student's *t* for *P* = 0.05 and *n*– 1 degrees of freedom. *N* is the total number of transects that could fit into the whole reef area of Kimbe Island. *s* (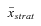) represents the standard deviation of overall stratified mean density (i.e., standard error). Other terms are defined in Table 1

Population estimate	
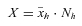	1688
Variance of stratified mean	
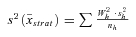	0.123
95% confidence limits	
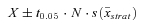	310

### 2007 Juvenile collections

A total of 443 juvenile *A. percula* were collected from the three study areas: 161 from Kimbe Island, 22 from the Garua group, and 260 from the Cape Heusner group. A total of 159 *C. vagabundus* recruits were collected from the three study areas: 103 from Kimbe Island, 31 from the Garua group, and 25 from the Cape Heusner group.

### Parentage analysis

**Figure 2:**
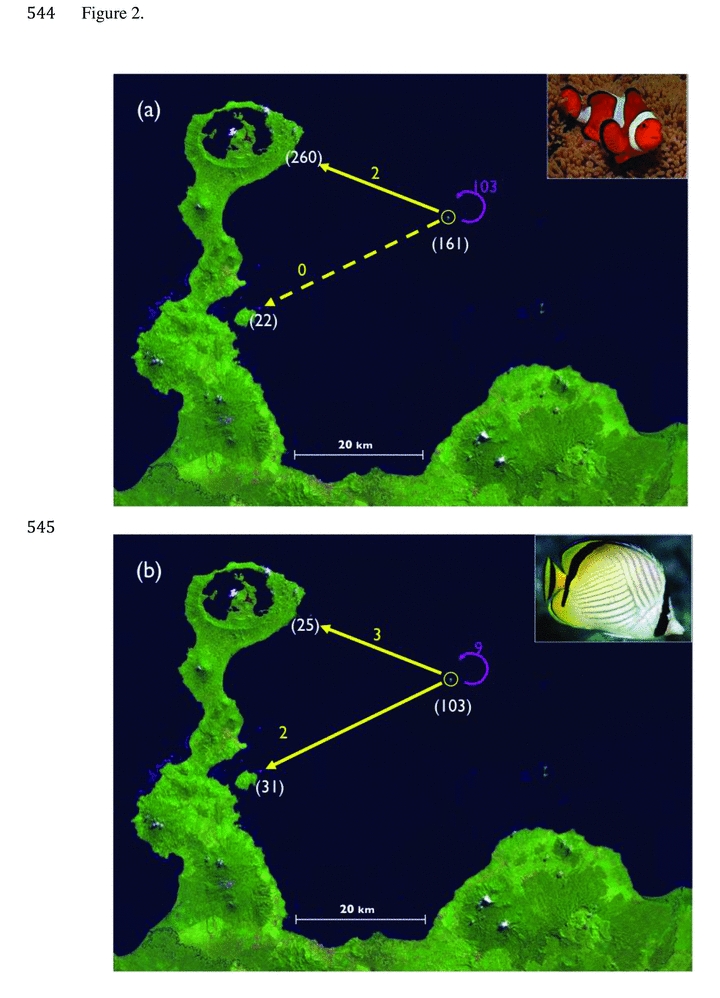
Natal origin and settlement patterns of recruits of (A) *Amphiprion percula* and (B) *Chaetodon vagabundus* among three reef groups in Kimbe Bay, Papua New Guinea in 2007. White numbers in parentheses indicate the total number of recruits screened for parentage using microsatellite markers against a pool of potential parents from Kimbe Island. Purple arrows indicate recruits that settled on the same reef as their parents at Kimbe Island (i.e., self-recruitment). Yellow arrows indicate recruits that were born at Kimbe Island that dispersed as pelagic larvae to Garua or Cape Heusner. The adjacent number in the corresponding color of an arrow indicates the number of recruits with that origin and settlement pattern, as determined by parentage analysis.

**Figure 3:**
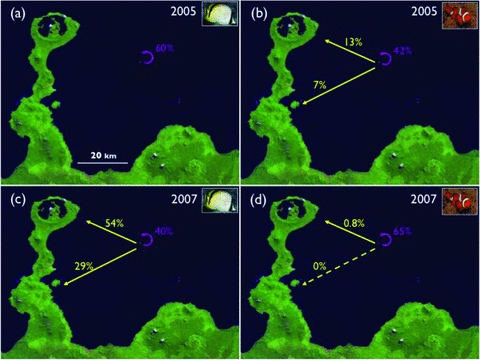
Comparison of self-recruitment and dispersal patterns for two species over time in Kimbe Bay, Papua New Guinea. Purple text indicates the percentage of recruits at Kimbe Island that originated from parents at Kimbe Island (i.e., self-recruitment). Yellow text and arrows indicate the percentage of recruits at Garua and Cape Heusner that were born at Kimbe Island and dispersed as pelagic larvae to these areas. (A) Self-recruitment measured for *Chaetodon vagabundus* at Kimbe Island in 2005 (Almany et al. 2007). (B) Self-recruitment and dispersal for *Amphiprion percula* in 2005 (Planes et al. 2009). (C) Self-recruitment and dispersal for *C. vagabundus* in 2007. Note that percentages are scaled to account for the fact that an estimated 22% of all adults at Kimbe Island were sampled. (D) Self-recruitment and dispersal for *A. percula* in 2007.

Of the 447 juvenile *A. percula* screened for DNA parentage analysis, 111 were assigned to parents from Kimbe Island (Fig. 2A). At Kimbe Island, 103 of 161 (64.0%) juveniles were assigned to Kimbe Island parents (Figs. 2A, 3D). At Garua, no juveniles from a total of 22 were assigned to Kimbe Island parents, and at Cape Heusner, only two of 260 (0.8%) juveniles were assigned to Kimbe Island parents (Figs. 2A, 3D).

Of the 159 juvenile *C. vagabundus* screened, 14 were assigned to parents on Kimbe Island (Fig. 2B). Estimates of self-recruitment at Kimbe Island and the percentage of recruits at Garua and Cape Heusner derived from Kimbe Island were adjusted to account for that fact that only 22.1% (95% CI = 18.7–27.1%) of the total adult population at Kimbe Island was screened. As a result, while nine of 103 (8.7%) juveniles at Kimbe Island were assigned to parents at Kimbe Island (Fig. 2B), the scaled estimate of self-recruitment is 39.5% (Fig. 3C) (95% CI = 32.2–46.7%). At Garua, two of 31 (6.5%) juveniles was assigned to Kimbe Island parents, and at Cape Heusner, three of 25 (12.0%) juveniles were assigned to Kimbe Island parents (Fig. 2B). Scaling these results indicates that 29.2% (95% CI = 23.8–34.5%) of juveniles at Garua and 54.3% (95% CI = 44.3–64.2%) of juveniles at Cape Heusner dispersed to these sites after being spawned at Kimbe Island (Fig. 3C).

### Results from previous studies

Our results are most interesting when placed in the context of results from two previous studies in Kimbe Bay. Almany et al. (2007) used transgenerational isotopic labeling (TRAIL) (Thorrold et al. 2006) to estimate ≍ 60% self-recruitment for *C. vagabundus* at Kimbe Island in 2005 (data presented in Fig. 3A). Planes et al. (2009) used TRAIL to validate genetic parentage analysis (the same method employed in the present study) as a tool to examine self-recruitment at Kimbe Island as well as larval connectivity between Kimbe Island and other sites within Kimbe Bay for *A. percula* in 2005 (data presented in Fig. 3B). Planes et al. (2009) documented high levels of self-recruitment (42%) at Kimbe Island and that the Kimbe Island population produced larvae that successfully dispersed to other populations 15–35 km away.

## Discussion

The results of our study provide unique insight into how larval dispersal and self-recruitment varies among species and through time. We found broad agreement between our results and predictions based on reproductive mode and PLD of the study species. Higher self-recruitment rates and lower connectivity were associated with benthic spawning and a relatively short PLD in *A. percula* compared to *C. vagabundus*. However, we also noted significant variability through time for both species that argued against a purely biological explanation for population connectivity in Kimbe Bay. Other factors, presumably including local current patterns, must also influence larval dispersal, to some degree.

Results from 2007 confirmed our earlier work that self-recruitment levels are remarkably high for *A. percula* and *C. vagabundus* at Kimbe Island (Almany et al. 2007; Planes et al. 2009), and remain relatively stable despite variability in connectivity patterns from Kimbe Island to other sites in Kimbe Bay. While there were some differences in levels of self-recruitment between 2005 and 2007, approximately 50% of the juveniles collected were spawned by parents on Kimbe Island reefs for both species. The remaining juveniles presumably were spawned on reefs at least, and likely considerably further than, 10 km from Kimbe Island; the nearest reef to Kimbe Island is 10 km distant, but is poor habitat for both species. High rates of self-recruitment in *A. percula* is perhaps not surprising as adults spawn benthic eggs that hatch as larvae with PLDs of less than two weeks, as suggested by Planes et al. (2009). Anemonefish larvae also possess significant locomotory and sensory abilities that may help them avoid dispersal away from the sensory halo of Kimbe Island (Fisher et al. 2000; Dixson et al. 2008). While obviously still significant (∼40%), self-recruitment levels in *C. vagabundus* were 20% lower than in *A. percula* for 2007 based on DNA parentage estimates. This difference probably reflects, at least to some degree, the fact that adult *C. vagabundus* spawn pelagic eggs and larvae that spend four to seven weeks in pelagic habitats before settling onto reefs. Nonetheless, the observation that populations of both species occupying a small reef (0.47 km^2^) on an oceanic island relied upon a significant amount of local replenishment has clear implications for the efficacy of MPAs. High levels of self-recruitment may be an important mechanism underlying the rapid increases in abundance and biomass observed in some MPAs (e.g., Roberts 1995; McClanahan et al. 2007; Russ et al. 2008). Certainly a population replenishment strategy that includes a balance between self-recruitment and long-distance dispersal would serve to maximize the potential for long-term population persistence; self-recruitment facilitates long-term population persistence (Hastings and Botsford 2006) while long-distance dispersal contributes to the maintenance of genetic diversity and ensures multiple pathways to recovery from disturbance.

Patterns of larval dispersal varied among years and between our two study species. We predicted that *C. vagabundus* larvae would be more likely to disperse among study sites than *A. percula* based on early life-history characteristics of the two species described above. Although limited to one sampling period, we did indeed detect greater proportional recruitment input from Kimbe Island to locations on the western shore of Kimbe Bay in *C. vagabundus* than in *A. percula*. Kimbe Island was the source of an estimated 29% and 54% of *C. vagabundus* recruits at Garua and Cape Heusner, respectively, in 2007. In contrast, during this same period Kimbe Island was the source of less than 1% of the *A. percula* juveniles collected at either Garua or Cape Heusner. However, the magnitude of dispersal varied through time, with Kimbe Island supplying as much as 13% to as little as none of the juveniles to these distant reefs over two years. Whether this degree of connectivity is sufficient to maintain *A. percula* populations remains to be answered, and will depend on intrinsic factors including the rate of natural mortality and extrinsic factors such as removal rates from disturbances including fishing, collecting, or other perturbations. Nonetheless, it seems reasonable to assume that the higher level of connectivity observed in *C. vagabundus* would facilitate, at least to a greater degree than *A. percula*, population persistence and recovery from disturbance. Although no information about the physical environment of Kimbe Bay is available at a spatial resolution matching our study data, the bay is known to have a hydrodynamic regime characterized by intermittent eddies originating from instabilities in the South Equatorial Current and New Guinea Coastal Current (Steinberg et al. 2006). We intend for future work to incorporate an assessment of fine-scale hydrodynamics to better understand the role that oceanography plays in larval dispersal patterns of these species.

It remains difficult to assess the generality of the dispersal patterns that we found in Kimbe Bay as there are few data available on other species or at other locations with which to compare our results. In earlier work we found self-recruitment levels of ≍ 32% in panda clownfish (*A. polymnus*) for a single, isolated metapopulation occupying 0.02 km^2^ in Kimbe Bay (Jones et al. 2005). Overall self-recruitment was significantly lower (18%) in a metapopulation of the panda clownfish occupying a much larger area along the southern coast of Papua New Guinea, with significant levels of connectivity among subpopulations separated by 2–28 km (Saenz–Agudelo et al. 2011). Christie et al. (2010b) suggested that there must be significant levels of self-recruitment in a benthic spawning damselfish (*Stegastes partitus*) based on several DNA matches between juveniles and adults. Most recently, Christie et al. (2010a) documented dispersal distances of up to 184 km in a pelagic spawning surgeonfish *Z. flavescens* without detecting any evidence for self-recruitment. Both are certainly consistent with the hypothesis that self-recruitment is likely to be higher in benthic spawning species than in pelagic spawning species (see also Weersing and Toonen 2009; Reginos et al. 2011), although our current results suggest that self-recruitment is a critical process for both of our study species within Kimbe Bay.

While many questions about larval dispersal and population connectivity remain unanswered, genetic parentage analysis is clearly a powerful technique to test the effectiveness of MPA networks and to verify results from studies that employ indirect methods of estimating these parameters including coupled biophysical models (e.g., Paris et al. 2007) and geochemical analysis of otoliths (Elsdon et al. 2008). Furthermore, assuming that our results can be extended to other species and locations (admittedly, an assumption that remains to be tested), there is good reason to be optimistic about the efficacy of MPAs as management tools. This study provides support for the argument that individual MPAs can work to increase the abundances of fish species within MPAs, regardless of species’ life histories (given appropriate initial population size and protection). In light of our findings, greater attention must be given to considerations of larval connectivity to enhance the resilience and recovery of populations within a MPA network in response to disturbance. We found clear and consistent differences in realized dispersal between a benthic and pelagic spawning species, with the latter displaying greater connectivity. Whether such patterns are temporally or spatially consistent between these two dominant fish life histories is unclear, highlighting the need for an increased number of studies generating this type of data. If consistent differences in connectivity do occur as general rules between spawning modes, the goals of any given MPA network must be explicitly defined to determine the optimum location, size, and spacing among the constituent parts of the network for particular taxa.
